# Studying carrier frequency of spinal muscular atrophy in the State of Qatar and comparison to other ethnic groups: Pilot study

**DOI:** 10.1002/mgg3.2184

**Published:** 2023-11-15

**Authors:** Faisal Ibrahim, Dinesh Velayutham, Mohamed Alsharshani, Usama AlAlami, Manar AlDewik, Tala Abuarja, Hilal Al Rifai, Nader I. Al‐Dewik

**Affiliations:** ^1^ Diagnostic Genetics Division (DGD), Department of Laboratory Medicine and Pathology (DLMP) Hamad Medical Corporation (HMC) Doha Qatar; ^2^ Liberal Arts and Science Hamad Bin Khalifa University (HBKU) Doha Qatar; ^3^ School of Life Science Manipal Academy of Higher Education (MAHE) Dubai UAE; ^4^ Department of Research and Translational and Precision Medicine Research Lab Women's Wellness and Research Center, Hamad Medical Corporation Doha Qatar; ^5^ Department of Pediatrics and Neonatology, Neonatal Intensive Care Unit, Newborn Screening Unit, Women's Wellness and Research Center Hamad Medical Corporation Doha Qatar; ^6^ Translational Research Institute (TRI) Hamad Medical Corporation (HMC) Doha Qatar; ^7^ Genomics and Precision Medicine (GPM), College of Health & Life Science (CHLS) Hamad Bin Khalifa University (HBKU) Doha Qatar; ^8^ College of Health and Life Sciences Hamad Bin Khalifa University, Education City Doha Qatar

**Keywords:** copy number (CN) variations, SMA, *SMN1*, *SMN2*, SNPs

## Abstract

**Background:**

Spinal muscular atrophy (SMA) is an autosomal recessive disease caused by mutations and deletions in *SMN1* at exon 7. The carrier frequency for *SMN1* mutations ranges from 2 to 4% in the general population.

**Methods:**

We examined allelic, genotypic relatedness and copy number (CN) variations and frequencies of *SMN1* and *SMN2*, in 13,426 samples from Qatar biobank (QBB) to provide a precise estimation of SMA carrier frequency in Qatar in comparison to other populations.

**Results:**

The SMA carrier frequency was found to be (2.8%) and the rs143838139 was found in 491/13426 (3.66%) of individuals. The SNP rs121909192, which is a pathogenic risk factor, was found in 321/13500 (2.38%). In Addition 242/11379 (2.13%) had two copies of *SMN1* and the rs143838139, which may explain the (2 + 0) silent carrier. Additionally, two participants were found to be SMA type 4 with 0 and 4 copy numbers in *SMN1* and *SMN2*, respectively.

**Conclusion:**

The SMA carrier frequency in Qatar was found to be comparable to Saudi Arabia and Caucasians. The likely pathogenic variant, rs121909192, was found to be significantly higher when compering with other in our study. The rs143838139 variant, which has a strong association with the silent carrier genotype, has been found. Consequently, testing for this SNP may enhance the precision of evaluating the likelihood of a patient having an affected child. We conclude that the frequency of SMA carriers varies within the Qatar population and other ethnic groups.

## Graphical abstract

Spinal muscular atrophy (SMA) is an autosomal recessive disease caused by mutations and deletions in SMN1 at exon 7. We examined allelic, genotypic relatedness and copy number (CN) variations and frequencies of SMN1 and SMN2, in 13, 426 samples from Qatar biobank (QBB) to provide a precise estimation of SMA carrier frequency in Qatar in comparison to other populations. The SMA carrier frequency was found to be (2.8%), and the rs143838139 was found in 491/13426 (3.66%) of individuals.

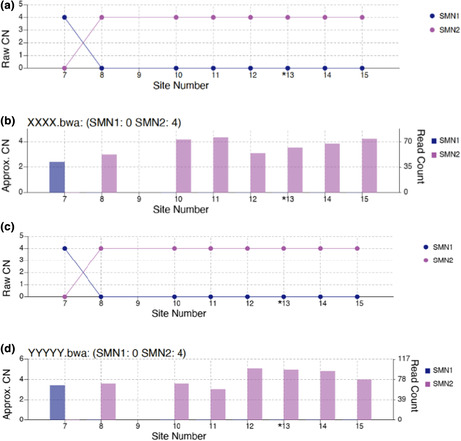

## 1 INTRODUCTION

Spinal muscular atrophy is an autosomal recessive neuromuscular disease characterized by degeneration of the anterior horn cells of the spinal cord, leading to progressive symmetrical limb and trunk paralysis associated with muscular atrophy (Prior, 2010). The incidence of SMA is 1 in 6000–10,000 live births, and the carrier frequency is 1:25–50 among different ethnic groups (Hendrickson et al., 2009; MacDonald et al., 2014; Ogino, Leonard, Rennert, Ewens, & Wilson, 2002; Sugarman et al., 2012). Its subtype (SMA I–SMA IV) is classified relative to the age of onset and severity of the disease (Lunn & Wang, 2008).

The survival motor neuron (SMN) gene is the main SMA determining gene and comprises *SMN1* and *SMN2* present on 5q13. *SMN1* and *SMN2* genes are typically distinguished by a single nucleotide difference (840 C > T) (Alías et al., 2009; Anhuf et al., 2003; Cusin et al., 2003; Feldkötter et al., 2002; Ogino, Leonard, Rennert, & Wilson, 2002). This single functional difference occurs at exon 7 (Al Jumah et al., 2022; Chen et al., 1999; Mailman et al., 2001, 2002). In most cases of SMA, individuals have a homozygous loss of function of the survival motor neuron gene *SMN1*, mainly due to a homozygous deletion of exon 7, with a much smaller quantity of residual SMN protein expression from each SMN2 copy. The number of *SMN2* copies varies within the general population and is inversely associated with disease severity as more *SMN2* copies ensures that the absolute amount of the SMN protein produced is higher (D'Amico et al., 2011), such that affected individuals with three or more copies of *SMN2* typically have milder forms of the disease (Al Jumah et al., 2022). Furthermore, it is now established that SMA is caused by deletions or intragenic mutations of *SMN1*. Homozygous deletion of *SMN1* is found in more than 92% of SMA patients, and deleteriously mutated in the remaining patients (Lyahyai et al., 2012; Wirth, 2000). In those remaining patients, small mutations that abolish the production of the SMN protein are found, mostly in combination with an *SMN1* deletion (~4%) (Sugarman et al., 2012; Verhaart et al., 2017).

Testing for SMA is of great importance and through carrier screening the identification of asymptomatic carrier couples with no family history at risk of transmitting a genetic disease to their future offspring is possible. The American College of Medical Genetics (ACMG) recommended routine carrier screening for SMA in the general population because of its high carrier frequency and the severity of the genetic disease (Kraszewski et al., 2018; Mailman et al., 2002; Prior et al., 2010). Carrier frequency for SMA ranges depending on ethnicity (Lyahyai et al., 2012; Ogino, Leonard, Rennert, Ewens, & Wilson, 2002; Prior et al., 2010; Sugarman et al., 2012). In Qatar, the premarital clinic issued a compulsory medical examination prior to marriage, by the Emiri decree in 2009, requiring all couples of Qatari citizens and residents to test for various diseases/disorders including genetic diseases such as homocystinuria, cystic fibrosis, and the option of SMA; such tests are performed at any genetic testing clinic in Qatar (Al‐Dewik et al., 2018).

The utility of population‐wide carrier screening has been demonstrated in pilot studies (Mailman et al., 2002). The key to screening for SMA is (1) determining the copy number of *SMN1* for SMA diagnosis and carrier testing and (2) determining the copy number of *SMN2* for clinical classification and prognosis. Traditionally, SMA testing, and carrier testing are done with polymerase chain reaction (PCR)‐based assays, such as quantitative PCR (qPCR) (Kraszewski et al., 2018) multiplex ligation‐dependent probe amplification (MLPA), and digital PCR (Anhuf et al., 2003; Arkblad et al., 2006; Ashley, 2015; Feldkötter et al., 2002; Huang et al., 2007; Kubo et al., 2015; Scarciolla et al., 2006; Stabley et al., 2015; Sutomo et al., 2002; Tomaszewicz et al., 2005; van der Steege et al., 1995; Zhong et al., 2011). Such methods primarily determine the copy number of *SMN1* based on the c.840C > T site that differs between *SMN1* and *SMN2*. With recent advances in next‐generation sequencing (Chen et al., 2020), it is now possible to profile a large number of genes or even the entire genome at high throughput and in a clinically relevant timeframe. Driven by these advances, many countries are currently undertaking large‐scale population sequencing efforts (Labrum et al., 2007; Turnbull et al., 2018). One of the most robust methods to detect SMA carriers is through whole genome sequencing where *SMN1* and *SMN2* copy numbers can be determined (Labrum et al., 2007; Turnbull et al., 2018). Sequencing across the whole genome and using different SNPs alleles and the identification of genotypes can improve carrier detection and provide more accurate estimates of residual risk with respect to SMA carrier status (Labrum et al., 2007).

Population‐specific studies on SMA provide insufficient information to support the calculation of carrier frequencies and risk assessments due to widespread carrier screening methods (Chen et al., 2005, 2020). Consequently, carrier screening must be performed by sensitive methods that can distinguish *SMN1* from *SMN2* (Turnbull et al., 2018). Therefore, we performed a wide SMA carrier genetic screening study using whole genome sequencing results from QBB to assess the prevalence of *SMN*1 deletions, carrier frequency, and the frequency and combined impact of the *SMN*1 and *SMN*2 copy number (CN) in a study consisting of 11,383 healthy individuals from the state of Qatar and comparing our results to the carrier results of other population including Caucasians, Europeans, Africans, East Asians, South Asians, and admixed Americans consisting of Colombians, Mexican‐Americans, Peruvians, and Puerto Ricans. The variants tested were rs143838139 (c.*3 + 80 T > G), a variant associated with a haplotype specific for SMN1 duplication alleles, rs141760116 (c.835‐2A > G), a splice acceptor site at SMN1 and two missense *SMN1* variants rs1554066397 (c.5C > G) and rs121909192(c. 859G > C); all variants have previously been reported in association with cases of SMA on ClinVar.

## 2 MATERIALS AND METHODS

### 2.1 SMN copy number caller

The SMN copy number caller tool is capable of characterizing both whole‐gene deletions/duplications and partial deletions of a region that includes exon 7 and 8 and can detect small variants linked to silent SMA carriers where two copies of *SMN1* are found on the same haplotype (c.*3 + 80TG / g.27134 T > G). It was employed in this study to determine the copy number of full‐length *SMN*1, full‐length *SMN*2, as well as *SMN*2 delta7–8 (*SMN*2 with a deletion of Exon7‐8) from 13,426 out of 14,664 whole‐genome sequencing (WGS) BAM files from the Qatar Biobank (QBB) participants (1238 results did not pass the quality control (QC) test and were omitted from our analysis).

This caller is designed to work with standard WGS sequencing depth (≥30X). It works by initially counting aligned reads to *SMN1* or *SMN2*. Any read counts in the 22.2 kb region (exons 1–6) are used to calculate total SMN CN (*SMN1, SMN2* and SMN2*∆7–8)*, while read counts in the 6.3 kb region (exons 7–8) are used to calculate the CN of intact SMN (*SMN1* and *SMN2*); the truncated SMN CN (SMN2*∆7–8)* is caclulated by subtracting the intact SMN CN from the total SMN CN.

After calculating the summed copy number, we differentiated *SMN*1 from *SMN*2 using supporting read counts at base differences between *SMN*1 and *SMN*2. The individual CN of *SMN1* or *SMN2* at each site is calculated by considering the summed SMN CN and the fraction of *SMN1* or *SMN2* supporting reads out of all *SMN*1 + *SMN*2 reads. During the development of the caller, we called the CNs of *SMN*1 and *SMN*2 at the 16 different sites that were extracted from the reference genome, in the 1000 Genomes Project (1kGP) samples, and determined if the CN calls for each position were concordant with the CN calls at the c.840C > T or g.27134 T > G or rs143838139 splice variant site.

### 2.2 Visualizing the caller results

A visualization tool for producing dynamic representations of data and calls' QC was also created where the summed CNs of total (exons 1–6) and intact (exons 7–8) SMN (*SMN*1 + *SMN*2) are displayed against the population distribution.

The CN of *SMN*2 delta 7–8 is represented by the difference between the total and intact SMN CN. Individual CNs of *SMN1* and *SMN2* are determined using the sum of the intact CN and supporting read counts at eight base changes between *SMN*1 and *SMN*2.

### 2.3 SMA SNPs allele and genotype

The allelic and genotypic results of our individuals for rs121909192, rs1554066397, rs141760116, and rs143838139 were extracted from the QBB records. The rs121909192, rs1554066397, rs141760116, are reported as pathogenic/likely pathogenic, whereas the rs143838139 is a benign and known to be associated with silent carriers (2 + 0). The list of SNPs that was screened is shown in (Supplementary table 1)

## 3 RESULTS

### 3.1 *SMN1* CN: *SMN2* CN percentage

This is a population‐based study designed to record data obtained from whole genome sequencing of the Qatar biobank (QBB) participants with the aim to gain insights into the genetic architecture of a clinically relevant disease and its carrier frequency in the Middle Eastern Qatar population. The present study is based on whole genome sequencing data obtained from 13,426 participants of QBB. The results of *SMN1* CN: *SMN2* CN amongst all carrier and non‐carrier study individuals are shown in (Table 1). Carriers were defined as having only one copy of the *SMN1* gene. A total of 381/13426 individuals (2.8%) were identified as SMA carriers. The carrier rate was 2.8% indicating that the carrier prevalence was approximately 1:35 in the current study. The two copies of *SMN1* CN are seven times more prevalent (11379/13426) than the three copies of *SMN1* CN (1547/13426) with a frequency of 84.78% and 11.52 respectively. There were eight SMA carriers with one copy of *SMN1* and no copies of *SMN2* “1:00” (Table 1).

**TABLE 1:** Observed *SMN1* CN: *SMN2* CN in all study individuals (*n* = 13,426).

*SMN1* CN: *SMN2* CN	No of subjects	Interpretation	Percentage
4:0	56	Noncarrier	0.42
4:1	31	Noncarrier	0.23
4:2	23	Noncarrier	0.17
4:3	1	Noncarrier	0.01
4:4	1	Noncarrier	0.01
3:0	241	Noncarrier	1.8
3:1	935	Noncarrier	6.96
3:2	313	Noncarrier	2.33
3:3	54	Noncarrier	0.402
3:4	3	Noncarrier	0.02
2:0	799	Noncarrier	5.6
2:1	3806	Noncarrier	28.35
2:2	6241	Noncarrier	46.48
2:3	504	Noncarrier	3.75
2:4	29	Noncarrier	0.22
1:0	8	SMA carrier	0.06
1:1	98	SMA carrier	0.73
1:2	195	SMA carrier	1.45
1:3	76	SMA carrier	0.57
1:4	4	SMA carrier	0.03

In our samples, the frequency of individuals with the SMA carrier “1:2” CN was the highest 195/13426 with a percentage of (1.45) compared with other individuals with the SMA carrier “1:1” 98/13426 and “1:3” 76/13426 with a percentage of 0.73% and 0.57% respectively (Table 1).The highest frequency of *SMN1* CN: *SMN2* CN was seen in individuals with the “2:2” CN 6241/13426 (46.48%) followed by individuals with the “2:1” 3806/13426 (28.35%) (Table 1).

**FIGURE 1:**
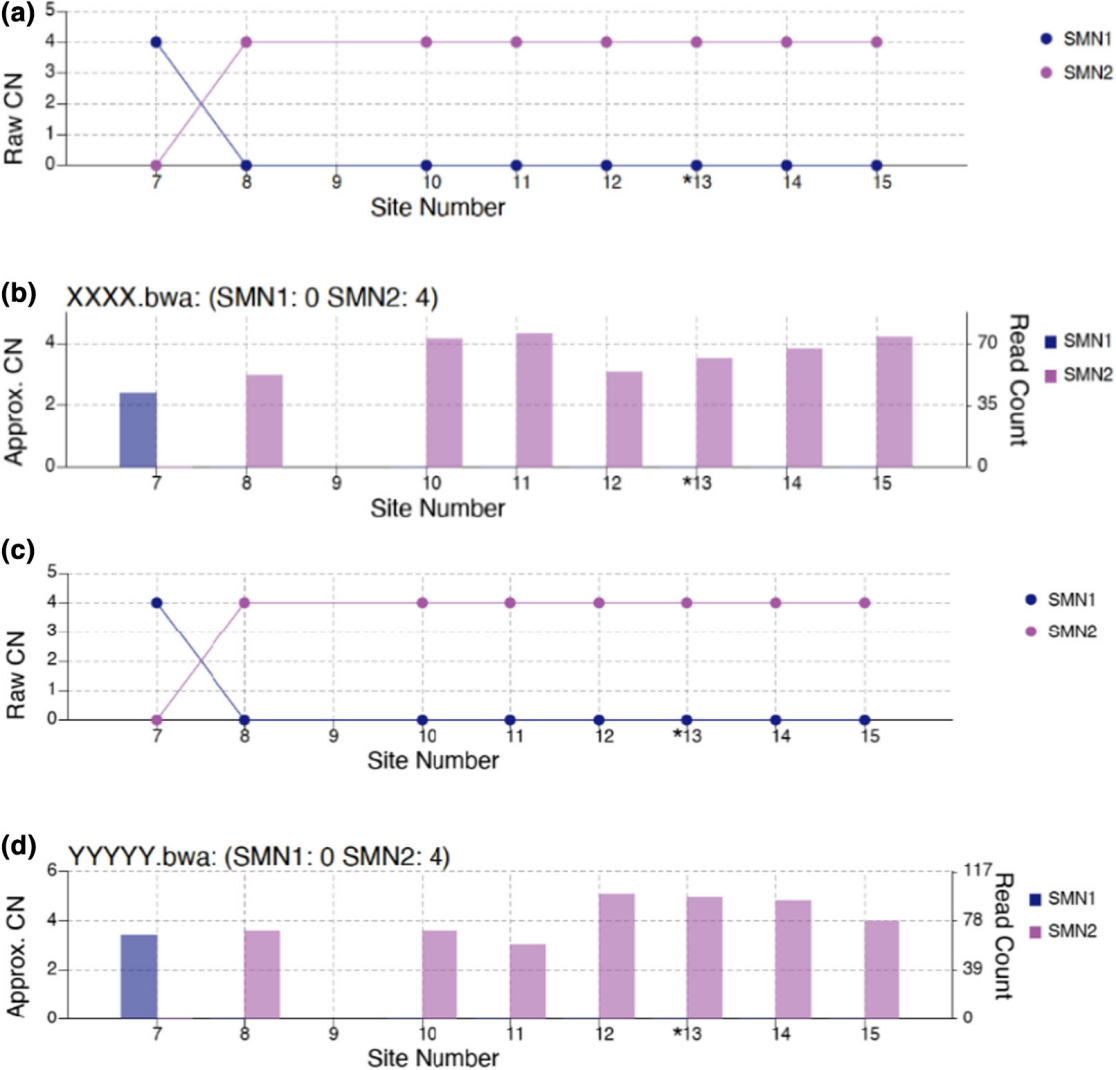
Visualization of SMN CN Caller result for two SMA type 4 cases. A + C. Raw CN values for *SMN1* and *SMN2* at 8 sites (site number 7–8 and 10–15). The raw CN of *SMN1* and *SMN2* at every site is calculated as the CN of intact SMN multiplied by the fraction of *SMN1/SMN2* supporting read counts by total supporting read counts. B + D. Raw read count is displayed on the right y axis and the left y‐axis depicts estimated CN found by dividing the read count by the median haploid depth of the sample.

Of note, four (0.03%) carrier individuals were found to have one *SMN1* CN and four *SMN2* CN “1:4” (Table 1). In addition, two participants were found to be SMA type 4 with 0 and 4 copy numbers in *SMN1* and *SMN2*, respectively (Figure 1).

Only one individual 1/13,426 had six copy number callers (CN) of the *SMN*1 gene, (Table 2).

**TABLE 2:** *SMN1* CN and *SMN2* CN amongst all study individuals (*n* = 13,426).

	No of subjects	Percentage
*SMN1* CN		
0	2	0.015
1	381	2.84
2	11379	84.75
3	1547	11.52
4	112	0.83
5	3	0.022
6	1	0.0074
*SMN2* CN		
0	1105	8.23
1	4870	36.27
2	6772	50.44
3	638	4.76
4	39	0.29
5	1	0.0074

### 3.2 Comparison of *SMN1* , *SMN2,* and *SMN2* delta7‐8 CN frequency

**FIGURE 2:**
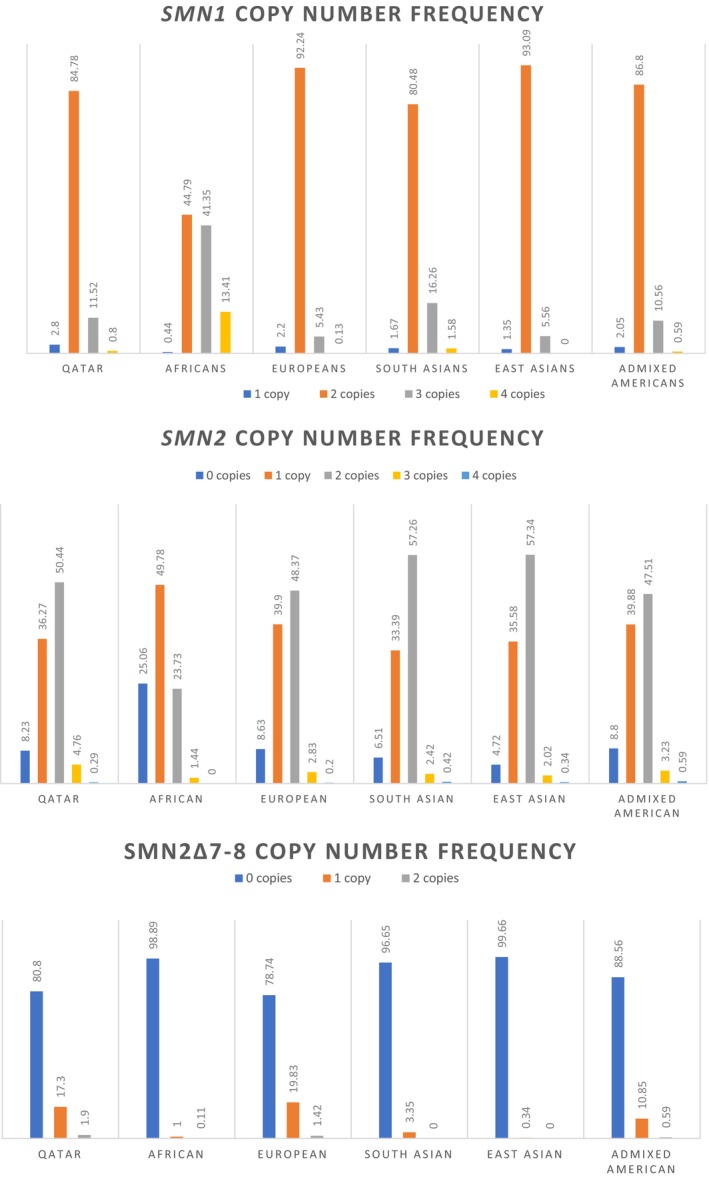
*SMN1*, *SMN2*, and *SMN2*∆7–8 CN frequency across populations in comparison to our cohort in Qatar. The comparison of CN frequency of populations in Africa (902 individuals), Europe (9648 individuals), South Asia (1199 individuals), East Asia (593 individuals), and Admixed America (341 individuals) as a percentage relative to the cohort in Qatar (14,208 individuals/13,426 individuals for *SMN2*∆7–8*)* (Adapted and reproduced data from Chen et al., 2020).

Our study results of *SMN1*, *SMN2*, and *SMN2* delta 7–8 copy number and frequencies and the comparison regarding to other populations is reported in (Figure 2).

In *SMN1*, 2 CNs were the most prevalent across all populations including Qatar (84.78%) while 2 CNs were most commonly reported in *SMN2* except in the African cohort with 1 CN of *SMN2*. Calculations of CN from the exon 7–8 region for the 13,426 samples where we identified instances of *SMN2* ∆7–8 included 10845 samples with zero copies (80.78%), 2320 samples with one copy (17.30%) and 255 samples with two copies (1.91%); other populations also reported a distinctly greater prevalence of 0 CN in SMN2 ∆7–8 relative to one and two copies.

### 3.3 Analysis for the g.27134 T > G polymorphism and detection of silent carriers

Our study detected the presence of the SNP (rs143838139), used to screen for potential silent carriers (2 + 0), which was found in 491/13,426 (3.66%) of all individuals and in 242/11379 (2.13%) of who had two copies of *SMN1* (Table  3).

**TABLE 3:** SMA SNP genotype and allele results of the Qatar population.

Genetic variants	Pathogenicity	Genotype	QBB Qatar	All^a^	*p* value	Allele	QBB Qatar	All^a^	*p* value
rs141760116 *N* = 14,666	Likely pathogenic	AA	14,661 (99.97%)	2202 (99.96%)	0.793112	A	29,327 (99.98%)	248,209 (99.999597%)	0.1599
		AG	5 (0.034%)	1 (0.04%)		G	5 (0.0173%)	1 (0.00041%)	
		GG	0 (0.0%)	0 (0.0%)					
rs143838139* *N* = 13,841	Benign	TT	13,276 (96.0%)	2069 (82.6%)	<0.00001	T	27,042 (97.69%)	130,634 (92.1%)	0.6649
		TG	491 (3.55%)	415 (16.4%)		G	639 (2.31%)	11,270 (7.9%)	
		GG	74 (0.53%)	20 (0.8%)					
rs1554066397 *N* = 1480	Pathogenic	CC	1479 (99.932%)	2062 (99.4%)	0.868	C	2959 (99.97%)	1278 (99.922%)	0.6649
		CG	1 (0.068%)	0 (0.0%)					
		GG	0 (0.0%)	12 (0.578%)		G	1 (0.03%)	1 (0.0782%)	
rs121909192 *N* = 13,500	Likely pathogenic	GG	13,179 (97.62%)	2500 (99.84%)	<0.00001	G	26,627 (98.62%)	5003 (99.90016%)	<0.00001
		GC	269 (1.99%)	3 (0.12%)		C	373 (1.38%)	5 (0.09984%)	
		CC	52 (0.39%)	1 (0.04%)					

^a^ Frequency results were obtained from Ensembl genome browser 108 https://www.ensembl.org.

### 3.4 SMA SNP genotyping results

SMA SNPs genotyping results of our tested individuals are listed in (Table  4).

**TABLE 4:** SMA SNP genotype results for rs143838139 and rs121909192 in Qatar population compared with different ethnicities.

Genetic variants	Genotype	Qatari Total no 13841 and 13,500	AFR^a^ Total no 661	AMR^a^ Total no 347	EAS^a^ Total no 504	EUR^a^ Total no 503	SAS^a^ Total no 489
rs143838139	TT	13,276 (96.0%)	250 (37.8%)	331 (95.4%)	504 (100.00%)	499 (99.2%)	485 (99.2%)
	TG	491 (3.55%)	391 (59.2%)	16 (4.6%)	^b^	4 (0.8%)	4 (0.8%)
	GG	74 (5.3%)	20 (3.02%)	^b^	^b^	^b^	^b^
rs121909192	GG	13,179 (97.62%)	611 (100.0%)	347 (100.0%)	^b^	501 (99.6%)	487 (99.6%)
	GC	269 (1.99%)	^b^	^b^	^b^	1 (0.2%)	2 (0.4%)
	CC	52 (0.39%)	^b^	^b^	^b^	1 (0.2%)	^b^

^a^ Results were obtained from Ensembl genome browser 108 https://www.ensembl.org.

^b^ Results were not detected.

The SNP rs121909192 was present in 321/13500 (2.38%). This SNP showed the strongest association as a pathogenic/risk factor of SMA and was associated with increased risk of occurrence of SMA (*p* < 0.00001) (Table  3). The SNP with the strongest association with SMA silent carriers' frequency in our study was found to be rs143838139 (*p* < 0.00001) which is significant to other ethnicities. Of the 14,666 individuals tested, the SNP rs141760116 was present in five individuals (0.034%) and absent in the remaining 14,661 individuals (99.96%). Comparison of our results to other ethnic populations is recorded in Tables 3 and  4.

## 4 DISCUSSION

To date, there are a few studies on the prevalence/ incidence of SMA, fewer of which are recent with most being carried out in Europe (Chen et al., 1999).

Studies have shown that SMA is predominant in Middle Eastern countries, Iran, Egypt, Pakistan, and Saudi Arabia, where consanguineous marriages are common (Ibrahim et al., 2012; Thareja et al., 2021). Consanguineous marriages may play a role in the increased prevalence of SMA among the Qatari population as first‐cousin marriages are present. Moreover, second and third‐degree marriages are also present in the state of Qatar. Consanguineous marriages were seen in 397/599 (66.2%) Qatari families, and first cousin groups account for 65% of the Qatari population (Ben‐Omran et al., 2020). It is worth noting that our study individuals were recruited from QBB, which contained data from both Qatari nationals and long‐term residents (≥15 years) of Qatar. This explains the results and prevalence that is seen in this study.

We analyzed the CN distributions and recorded CN calls for *SMN1* and *SMN2* and *SMN1* CN: *SMN2* CN using sequencing data from 13,426 samples from QBB and compared our results to other population (Europeans, Africans, East Asians, South Asians, and admixed Americans consisting of Colombians, Mexican Americans, Peruvians, and Puerto Ricans) (Table  3). Tables 1 and 2 show the number of individuals and percentage of *SMN1* CN and *SMN2* CN in addition to *and SMN1* CN: *SMN2* CN in our study population.

Moreover, Table 2 shows the number of carriers identified across our Qatar population. Our results calculated the probability of being an SMA carrier when an individual without a family history of SMA carries one *SMN1* copy.

We identified a total of 381 samples (2.84%) with *SMN1* carrier (less than two copies) and 1663 (12.38%) with *SMN1* gains (more than two copies). 5975 (44.50%) samples consisted of *SMN2* losses and 678 (5.04%) with *SMN2* gains (Tables 1 and 2). The highest one‐copy carrier rate was identified in specimens from the Caucasian group with a frequency of 1 in 37 samples (2.7%) (Anhuf et al., 2003); our results were comparatively similar to them (2.8%) with a frequency of almost 1 in 1:35 samples.

The results we recorded for SMA carriers' frequency was higher than those previously reported in other populations (Anhuf et al., 2003; Chen et al., 1999; Sugarman et al., 2012). (Figure  2), but similar to what was found in Saudi Arabia where their analysis showed the presence of one copy of the *SMN1* gene in 108 samples and two copies (normal) in 4090 samples resulted in a carrier frequency of 2.6% (Al Jumah et al., 2022). Analysis of 150 Moroccan newborns predicted a carrier frequency of approximately 1:25 (Lyahyai et al., 2012), which is higher than our results of a frequency of 1 in 37 samples. Results regarding other ethnicities found that Europeans have the highest carrier frequency at 2.2%, followed by admixed Americans (2.05%), South Asians (1.67%), and East Asians (1.35%). Africans have the lowest carrier frequency (0.44%), whereas the African group had significantly lower one‐copy carrier genotypes when compared with our results, at 1 in 225 (0.44%, *p* < 0.05) (Chen et al., 2020). Such results are currently the lowest reported *SMN1* carrier frequencies for any population or ethnic group (Table 4). It was also found that the 1.67% carrier frequency detected in the South‐Asian sample is higher than that reported in East‐Asian populations (1.35%) (Chen et al., 2020). Moreover, our results are close to those of Middle Eastern countries including Iran, Egypt, Pakistan, and Saudi Arabia (Al Jumah et al., 2022; Ibrahim et al., 2012).

It was found that for all reported ethnic groups, including our Qatar population study but excluding the African group, the two‐copy genotype was over seven times more prevalent than the three‐copy genotype group (Sugarman et al., 2012). This is consistent with all previously published data showing the two‐copy genotype to be predominant. Surprisingly, the African population departed significantly from this genotype distribution, revealing similar frequencies for the two‐ and three‐copy genotypes (44.79% and 41.35% respectively) (Sugarman et al., 2012)., suggesting a much higher frequency of alleles with two or more *SMN1* copies relative to Qatar individuals and other ethnic groups (Table 4).

The most common combinations of *SMN1*:*SMN2* copy number seen in our results are 2:2 (46.48%) followed by 2:1 (28.35%). In general, individuals have more copies of *SMN1* than *SMN2*. The most common combinations of *SMN1*:*SMN2* copy number in other ethnicities are 2:2 (44.9%) and 2:1 (33.4%). excluding the Africans that show higher variability in both *SMN1* and *SMN2* CN. This observation is consistent with what was found in our results. The variability of *SMN1* copy number is much lower than that of *SMN2* copy number. Conversely, 54.76% of Africans have three or more copies of *SMN1*, which is more than double of what is observed in any of the other four populations and in Qatar. Africans also have significantly lower *SMN2* CN than the other populations.

We reported the frequency of the exon 7–8 deletion (*SMN2* delta7‐8) across our population compared with other populations in (Table  3). In our study, 17.3% had at least one copy of *SMN2* delta7‐8. It was also found that 21.25% of Europeans and 11.44% of admixed Americans have at least one copy of *SMN2* delta7‐8, while the frequency is lower in South Asians (3.35%), Africans (1.11%), and East Asians (0.34%)   (Figure  2) (Chen et al., 2020).

The g.27134 T > G SNP (rs143838139) is most strongly associated with two‐copy *SMN1* alleles and is implemented for the detection of the g.27134 T > G “2 + 0” silent carrier where one chromosome carries two copies of *SMN1* (either by *SMN1* duplication or gene conversion of *SMN2* to *SMN1*), and the other chromosome has no copies of *SMN*1. In our study, the rs143838139 SNP was found in 491 of the studied individuals (3.55%), and the silent carrier frequency was 242/11379 (2.13%) and absent in the remaining 13,350 individuals (96.45%) (Table 4). Based on the previous ethnic results, we found that the linkage of this SNP was the highest among African 59.2% (Table  4). Only 0.8% of individuals of Europeans and South Asian were carriers of the rs143838139 SNP (Table 4). This SNP is most strongly associated with two‐copy *SMN1* alleles in Africans. The preponderance of the two‐copy allele in the African group suggests a much higher frequency of individuals with the SMA silent carrier “2 + 0” genotype compared with other ancestries, where 33% of African individuals with two copies of *SMN1* also have the rs143838139 SNP (Sugarman et al., 2012).

The SNP rs121909192 was present in 321/13500 (2.38%) of our study population. This SNP showed the strongest association as pathogenic/risk factor with SMA and was associated with increased risk of occurrence of SMA. The SNP was most prevalent in the individuals of our cohort study. The SNP was present at relatively low levels in the European and South‐East Asian populations, 0.2% and 0.4%, respectively, and was absent in the African, American, and East‐Asian population (Table  4), which were limited in sample size compared with our Qatari study population. In further research, the use of a precision medicine strategy (Al‐Dewik et al., 2022; Al‐Dewik et al., 2019) and population health analyses (Zhai et al., 2023) should be taken into consideration for SMA.

## 5 CONCLUSION

In conclusion, the current research sheds light on the widespread of genetic diseases in our community and to the frequency of SMA carriers in Qatar. The SMA testing is highly recommended be done compulsory as part of premarital and/or genomic newborn screening. As a of premarital screening due to its high carrier rate in the community. DNA sequencing has become the preferred tool for testing disease‐causing variants throughout the genome. Large, multigene targeted sequencing panels are the future of clinical carrier testing, and there is a wide critical need for a comprehensive NGS test that includes SMA carrier detection worldwide. It is noteworthy to mention that our data are leaning toward the role of consanguineous marriages in causing this disease, but the limitation is that our study included individuals who are long‐term residents (≥15 years living in Qatar).This is a pilot study and future work to strengthen validity will be implemented through independent validation of CN and variants associated with SMA and silent carrier using both q‐PCR and microarray.

## AUTHOR CONTRIBUTIONS

**Nader I. Al‐Dewik:** Conceptualization, data curation, formal analysis, investigation, methodology, resources, validation, writing‐original draft, writing and editing review, supervision, funding acquisition, correspondence. **Faisal Ibrahim:** Writing and editing review, critical scientific review, formal analysis, and resources validation. **Dinesh Velayutham**: Formal analysis. **Mohamed Alsharshani:** Supervision. **Manar R. ALDweik:** Writing‐original draft, writing and editing review, and formal analysis. **Usama AlAlami:** Critical scientific and manuscript editing. **Tala Abuarja:** Manuscript editing and referencing.

All authors contributed to manuscript writing and proofreading. All authors have read and agreed to the published version of the manuscript.

## FUNDING INFORMATION

The publication of this article is funded by the Qatar National Library, Doha, Qatar.

## CONFLICT OF INTEREST STATEMENT

The authors declare no conflict of interest.

## INSTITUTIONAL REVIEW BOARD STATEMENT

This study was approved by the QBB.

## INFORMED CONSENT STATEMENT

Not applicable.

## Supporting information


**Table S1** List of SMA SNPs submitted to QBB and reported in ClinVar

## Data Availability

This is a research article, and all data generated or analyzed during this study are included in this published article [and its supplementary information files]. All enquiries should be directed to the corresponding author's Email: naldewik@hamad.qa; nader.al-dewik@kingston.ac.uk.
