# Supplementary material for: Studying carrier frequency of spinal muscular atrophy in the State of Qatar and comparison to other ethnic groups: Pilot study
Source: Mol Genet Genomic Med. 2023 Nov 15;11(12):e2184. doi: 10.1002/mgg3.2184 (PMC10724519; doi:10.1002/mgg3.2184)
Supplement: Supplementary file 1 — Table S1 List of SMA SNPs submitted to QBB and reported in ClinVar [file MGG3-11-e2184-s001.docx]

**Table S1:** List of SMA SNPs submitted to QBB and reported in ClinVar

| Chromosome | Position | ID |
| --- | --- | --- |
| Chrom 5 | 70925124 | rs1267335967 |
| Chrom 5 | 70925147 | rs1245780296 |
| Chrom 5 | 70925151 | rs1561498701 |
| Chrom 5 | 70938841 | rs1554081948 |
| Chrom 5 | 70938841 | CM108223 |
| Chrom 5 | 70938890 | rs1554081951 |
| Chrom 5 | 70941443 | rs796462131 |
| Chrom 5 | 70942359 | CM073366 |
| Chrom 5 | 70942481 | rs77668214 |
| Chrom 5 | 70942521 | rs75991011 |
| Chrom 5 | 70942752 | rs1561499720 |
| Chrom 5 | 70942799 | rs80145309 |
| Chrom 5 | 70942825 | rs1561499748 |
| Chrom 5 | 70944659 | rs1157575099 |
| Chrom 5 | 70944682 | rs1280965059 |
| Chrom 5 | 70944694 | rs1215894449 |
| Chrom 5 | 70944751 | CD145869 |
| Chrom 5 | 70944679 | rs1242763717 |
| Chrom 5 | 70951919 | rs1561503058 |
| Chrom 5 | 70951943 | rs1561503108 |
| Chrom 5 | 70951959 | rs1475940018 |
| Chrom 5 | 70951957 | rs1554082383 |
| Chrom 5 | 70946110 | rs1561500847 |
| Chrom 5 | 70951992 | rs763144752 |
| Chrom 5 | 70951962 | rs1179910122 |
| Chrom 5 | 70952074 | rs143838139 |
| Chrom 5 | 70952645 | rs200800214 |
| Chrom 5 | 70049701 | rs1490073523 |
| Chrom 5 | 70049706 | rs1278777005 |
| Chrom 5 | 70049766 | rs1232696462 |
| Chrom 5 | 70063417 | rs1554066599 |
| Chrom 5 | 70069271 | rs1408167278 |
| Chrom 5 | 70076563 | rs762444457 |
| Chrom 5 | 70076573 | rs1270114068 |
| Chrom 5 | 70925108 | rs1554066397 |
| Chrom 5 | 70925116 | rs1259001921 |
| Chrom 5 | 70925113 | rs1446964734 |
| Chrom 5 | 70925119 | rs1208576707 |
|  |  |  |

| Chrom 5 | 70925125 | rs1221447932 |
| --- | --- | --- |
| Chrom 5 | 70925180 | rs1170466474 |
| Chrom 5 | 70951946 | rs1164325688 |
| Chrom 5 | 70942503 | rs1554081968 |
| Chrom 5 | 70951938 | rs772466166 |
| Chrom 5 | 70951942 | rs76163360 |
| Chrom 5 | 70946160 | rs1554082114 |
| Chrom 5 | 70925108 | rs1554066397 |
| Chrom 5 | 70938849 | rs1554081950 |
| Chrom 5 | 70946127 | rs1554066659 |
| Chrom 5 | 70946157 | rs104893922 |
| Chrom 5 | 70951939 | rs141760116 |
| Chrom 5 | 70938845 | rs104893930 |
| Chrom 5 | 70942357 | rs1561499612 |
| Chrom 5 | 70942367 | rs104893927 |
| Chrom 5 | 70942389 | rs77804083 |
| Chrom 5 | 70942503 | rs1554081968 |
| Chrom 5 | 70946163 | rs1554066666 |
| Chrom 5 | 70951946 | rs1164325688 |
| Chrom 5 | 70076545 | rs121909192 |
| Chrom 5 | 70067122 | rs1450194682 |
| Chrom 5 | 70049686 | rs1160605944 |
| Chrom 5 | 70049690 | rs75030631 |
| Chrom 5 | 70049695 | rs1337306286 |
| Chrom 5 | 70049696 | rs1408282671 |
| Chrom 5 | 70049698 | rs1182283499 |
| Chrom 5 | 70049701 | rs1244662996 |
| Chrom 5 | 70049703 | rs1439722209 |
| Chrom 5 | 70049713 | rs895571119 |
| Chrom 5 | 70049722 | rs1343835779 |
| Chrom 5 | 70049731 | rs1295551831 |
| Chrom 5 | 70049762 | rs1202831370 |
| Chrom 5 | 70070704 | rs1354905416 |
| Chrom 5 | 70076561 | rs1332921390 |
| Chrom 5 | 70076544 | rs777199497 |
| Chrom 5 | 70076555 | rs200653528 |
| Chrom 5 | 70076786 | rs145999618 |
| Chrom 5 | 70952233 | rs147714462 |
